# Dataset regarding access to information for persons with disabilities in Serbia

**DOI:** 10.1016/j.dib.2020.106309

**Published:** 2020-09-14

**Authors:** Djordje M. Kadijevich, Dejan Masliković, Bojan M. Tomić

**Affiliations:** a Institute for Educational Research, Belgrade, Serbia; b Ministry of Culture and Information of the Republic of Serbia, Belgrade, Serbia; c Institute for Multidisciplinary Research, University of Belgrade, Belgrade, Serbia

**Keywords:** Access to information, Bayesian estimations, Binary variables, Path modeling, Persons with disabilities, State regulations

## Abstract

Access to information is key for improving the position of persons with disabilities in society. Familiarity with state regulations regarding access to information could be influenced by communication with state authorities concerning the rights of persons with disabilities, especially access to information. Familiarity with these regulations and the specified communication with state authorities might be affected by a number of background variables, such as age and education completed. To clarify relations among these variables, which would enable state authorities and other relevant institutions to define and implement policies that might improve matters, there is a need to prepare and analyze appropriate datasets concerning them. This paper describes such a dataset, preliminary in nature, obtained from answers to part of a questionnaire administered to persons with disabilities living in Serbia. Persons with innate or acquired physical and/or sensory disability were included in the research. This dataset contains raw data of nine variables, as well as analyzed data of ten variables derived from most of the raw data. Besides correlative analyses, the dataset was previously analyzed using PLS (partial least squares) path modeling. To reuse the dataset, a path model with Bayesian estimations may be applied, whose outcomes for different model priors (prior distributions) may be compared to those of the PLS path modeling. The dataset also contains data of two variables that may be included in further research.

## Specifications Table

**Table:** 

Subject	Social Sciences
Specific subject area	Disability Studies
Type of data	Table
How data were acquired	The dataset was obtained from a preliminary research. It was based upon answers to part of a questionnaire administered to persons with disabilities across Serbia. Originally, it was written in the Serbian language. An English-language translation of this part is given in the Appendix. The dataset comprises eighty-nine records, because complete data on the variables analyzed in this dataset were available for only eighty-nine out of one hundred participants who completed the questionnaire distributed.
Data format	Raw, Filtered, Dichotomized, Analyzed
Parameters for data collection	Data collection concerned seven background variables (gender, age, disability type, educational level completed, employment status, professional competence, support service) and two variables regarding access to information for persons with disabilities (communication with state authorities, familiarity with state regulations).
Description of data collection	Data collection was accomplished through the mediation of unions and associations of persons with disabilities, who selected those who were invited to complete the questionnaire. They mostly did so using e-mail. When electronic communication was not available, the questionnaire was completed in the traditional way with the assistance of surveyors.
Data source location	Institution: Institute for Multidisciplinary Research, University of Belgrade Corresponding author: Bojan M. Tomić Email: bojantomic@imsi.rs City: Belgrade Country: Serbia
Data accessibility	http://doi.org/10.5281/zenodo.4025980
Related research article	D.M. Kadijevich, D. Masliković, B.M. Tomić, 2020. Familiarity with state regulations regarding access to information for persons with disabilities in Serbia. International Journal of Disability, Development and Education. https://doi.org/10.1080/1034912X.2020.1802646

## Value of the Data

• The dataset is beneficial for clarifying the relationships among background variables and key variables regarding access to information for persons with disabilities (A2IPwDs).
• The dataset will be useful to scholars dealing with disability studies, who may conduct similar research on A2IPwDs by searching for a path model that involves other exogenous or moderating variables. It could also be useful for those who study marginalized groups or access to (state/government) information.
• The dataset may be reused to examine a path model with Bayesian estimations, or to identify types of persons with disabilities regarding access to information.
• The findings obtained from the dataset may support state authorities and other relevant institutions in defining and implementing policies that improve the overall position of persons with disabilities in society.
• Although the dataset was obtained from preliminary research, it may be used as a role model for conducting a survey on a larger sample.

## 1 Data Description

Regarding access to information, familiarity with state regulations – which is key to improving the position of persons with disabilities in society (e.g., [1]) – could be influenced by communication with state authorities. To clarify relations among familiarity with these regulations, specified communication with state authorities, and other background variables that may affect them (e.g., age and education completed), there is a need to prepare and analyze appropriate datasets. The outcomes concerning these datasets would support state authorities and other relevant institutions in defining and implementing policies that could improve matters (i.e. living and working conditions for persons with disabilities).

This section describes such a dataset. The dataset is stored in one-sheet spreadsheet file. The sheet comprises a table with eighty-nine rows and twenty-one columns of data. The first nine contain raw data, the next ten contain analyzed data derived from most of these raw data, whereas the remaining two contain data (also derived from some of the raw data) that may be included in further research.

Each row represents individual responses regarding participant's gender, age, disability type, educational level completed (i.e. the highest level completed), employment status, state authorities communication, state regulations familiarity, professional competence, and support service. For seven analyzed variables representing the first seven features (source [2]), the following values were used (their values are given in parentheses):

• Gender (1- male, 2- female);
• Age (in years as whole numbers);
• Disability type (1- innate, 2- acquired);
• Educational level completed (1- lower or upper secondary, 2- post-secondary);
• Employment status (1- employed, 0- unemployed);
• State authorities communication (1- satisfactory or good, 0- missing or poor);
• State regulations familiarity (1- familiar, 0- unfamiliar).

The data that may be used in further research deal with the two last features mentioned above: professional competence and support service. For two variables representing these features, the following values were used:

• Professional competence (1-attained through regular schooling, 0-unattained or attained through other ways);
• Support service (1-this service can possibly be used, 0-otherwise).

Note that one datum for Support service is missing, which reduces the size of the sample to 88.

The correlations among the first seven analyzed variables revealed that some expected relationships were, in fact, missing. For example, it was supposed that Educational level completed was positively related to State authorities communication (i.e. communication with state authorities). Following such findings, Educational level completed was considered as a 3-value variable (1-lower-secondary, 2-upper-secondary, 3-post-secondary) and added to the dataset, but, again, the expected relationship in question was missing. Because of that, several binary variables regarding Age and Educational level completed were defined and examined through additional correlative analysis. This examination pointed out two of them (Aged 40–49 and Upper-secondary education completed) that could influence State authorities communication. This influence was confirmed through a PLS (partial least squares) path modeling [2]. Because of that, the values of these two binary variables were added to the dataset, which finally comprised ten columns (attributes or fields) concerning the variables analyzed. Descriptive statistics for these ten variables (attributes) are reported in Table 1.

**Table 1:** Descriptive statistics for ten variables analyzed (adapted from [2]).

## 2 Experimental design, materials, and methods

The values of the first seven variables given in Table 1 were derived from the data collected through a survey. To this end, a written questionnaire on access to information written in the Serbian language was administered. The raw data was obtained from the participants' answers to part of the questionnaire given in the Appendix.

Data collection was carried out through the mediation of unions and associations of persons with disabilities. These institutions selected whom to invite to complete the questionnaire. The survey included persons with physical and/or sensory disability.

One hundred participants completed the questionnaire. To this end, most of them used e-mail. When electronic communication was not available, it was done in the traditional way with the assistance of local surveyors.

Concerned with the validity of data being collected, all participants were requested to complete questionnaires themselves, without any help from the assistants, family members or medical staff. That is the primary reason for obtaining only one hundred questionnaires out of all those distributed.

The dataset comprises eighty-nine records (rows), because complete data on the analyzed variables used in this dataset were available for only eighty-nine out of one hundred participants. The application of the Runs test showed that the order of the excluded records should be considered random (*N* = 100, Number of runs = 18, *z* = –1.346, *p* = 0.178). Furthermore, an exclusion of eleven records (or 10% statistically) is acceptable if a 10% missing data cutoff is assumed [3].

Although a convenience sample was used, the values of personal background variables (e.g., educational level completed, employment status) were similar to those reported by other studies on persons with physical and/or sensory disabilities regarding their population in Serbia (e.g., [4]). This suggests that the applied sample could be considered as representative.

To analyze the data collected, the participants' answers were mostly expressed as dichotomous variables. To this end, the answers were grouped to just two levels. For Educational level completed, a distinction “lower or upper-secondary education *vs* post-secondary education” was used, for example.

To examine the dataset, a correlative design was applied. Following the results of correlative analyses, three variables were eventually added to the dataset, as explained in the previous Data Description section.

**Fig. 1:**
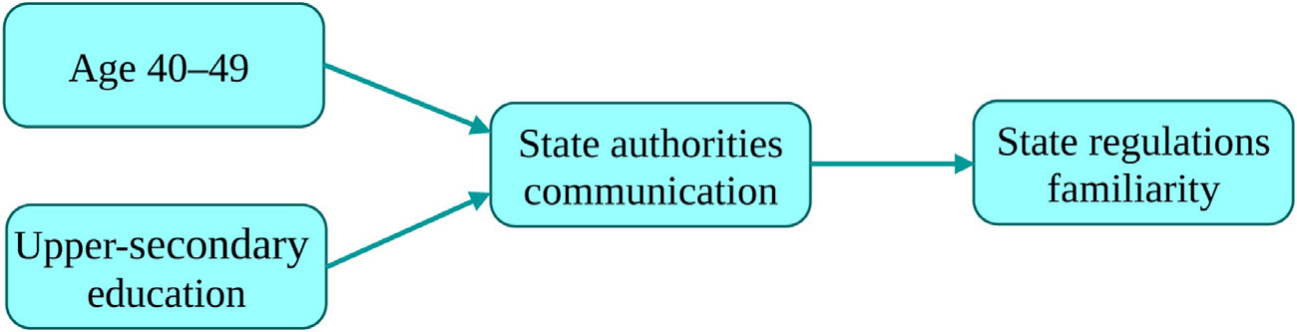
Path model examined (adapted from [2]).

Researchers examine indirect and direct effects among several independent and dependent variables in a simultaneous fashion by using path modeling (e.g., [5]). In doing that, they usually include variables with significant or almost significant correlations (e.g., *p* < 0.05, one-tailed) between them. The research study [2] associated with the dataset presented in this article examined the path model presented in Fig. 1, and in doing so SmartPLS software [6] was used.

**Fig. 2:**
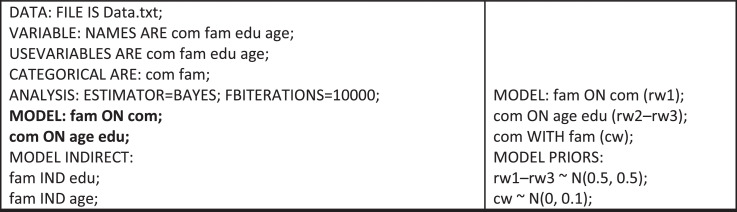
Mplus program code: full model with built-in priors (left); model section using user-defined priors (right).

To reuse the dataset, the path modeling may rely on Bayesian estimations, for example. To this end, Mplus software [7] may be used. By assuming that Data.txt, com, fam, edu, and age stand, respectively, for the dataset (as a CSV, comma-separated values, file), and variables State authorities communication, State regulations familiarity, Upper-secondary education completed, and Aged 40–49, the researcher could start with a model using built-in priors (prior distributions), such as that specified in Fig. 2 (left).

Then, its part given in bold might be replaced by statements referring to user-defined model priors (see Fig. 2, right), including the assumption of correlated residuals (statement com WITH fam). Instead of N(0.5, 0.5) and N(0, 0.1), other normal distributions might be appropriate, such as N(0.5, 0.25) and N(0, 0.01). Through experimenting with different distributions, the researcher may compare the findings of the Bayesian path modeling with those of the PLS path modeling reported in [2], taking into account the requirements for acceptable fit indices of Bayesian path models (e.g., [8]).

By considering variables Professional competence and Support service, the reuse of the dataset may be directed toward searching for a path model, which compared to the model presented in Fig. 1, includes additional or different variables.

## Ethics statement

The participants were informed that they had been chosen as representative persons for the needs of people with disabilities, that the survey was voluntary, that it would be anonymized, and that collected data would be used for scientific and practical purpose. They were also informed how they could get additional information about the questionnaire and the research conducted.

## CRediT authorship contribution statement

**Djordje M. Kadijevich:** Conceptualization, Methodology, Formal analysis, Writing - original draft. **Dejan Masliković:** Conceptualization, Investigation, Writing - review & editing. **Bojan M. Tomić:** Conceptualization, Writing - review & editing.

## Declaration of Competing Interest

The authors declare that they have no known competing financial interests or personal relationships which have, or could be perceived to have, influenced the work reported in this article.

## Appendix: Part of the questionnaire about access to information for persons with disabilities

*I. Socio-demographic characteristics*

1. Gender (check the box after the answer)
  1) Male
  2) Female
2. Age: _______ (insert years)
3. Completed education (check the box after the answer)
  1) Lower-secondary education
  2) Lower-secondary education (special school for students with disabilities)
  3) Upper-secondary education
  4) Upper-secondary education (special school for students with disabilities)
  5) College education
  6) Undergraduate education
  7) Graduate education (MS level)
  8) Graduate education (PhD level)
  9) Without formal education
4. Professional competence attained through (check the box after the answer)
  1) Regular schooling
  2) Professional rehabilitation
  3) Courses and the Internet
  4) Without professional competences
5. Employment status (check the box after the answer)
  1) Employed in your profession
  2) Employed outside your profession
  3) Unemployed
6. Disability type (check the box after the answer)
  1) Acquired
  2) Innate
10. Do you have the opportunity to use the support service? (check the box after the answer)
  1) Yes
  2) No

*IV. Communication with state authorities and public institutions*

16. How is your personal communication with state authorities? (mark the appropriate level)
  0- none
  1- weak
  2- satisfactory
  3- good
17. Are you familiar with state regulations regarding access to information for persons with disabilities in Serbia? (check the box after the answer)
  1) Yes
  2) No

## Research data for this article

The data described in this paper is deposited in Zenodo, http://doi.org/10.5281/zenodo.4025980.
